# Caffeic Acid Phenethyl Ester Inhibits Oral Cancer Cell Metastasis by Regulating Matrix Metalloproteinase-2 and the Mitogen-Activated Protein Kinase Pathway

**DOI:** 10.1155/2012/732578

**Published:** 2012-12-18

**Authors:** Chih-Yu Peng, Hui-Wen Yang, Yin-Hung Chu, Yu-Chao Chang, Ming-Ju Hsieh, Ming-Yung Chou, Kun-Tu Yeh, Yueh-Min Lin, Shun-Fa Yang, Chiao-Wen Lin

**Affiliations:** ^1^School of Dentistry, Chung Shan Medical University, Taichung 40201, Taiwan; ^2^Department of Dentistry, Chung Shan Medical University Hospital, Taichung 40201, Taiwan; ^3^Institute of Medicine, Chung Shan Medical University, Taichung 402, Taiwan; ^4^School of Medical Laboratory and Biotechnology, Chung Shan Medical University, Taichung 40201, Taiwan; ^5^Department of Pathology, Changhua Christian Hospital, Changhua 50006, Taiwan; ^6^Department of Medical Research, Chung Shan Medical University Hospital, Taichung 40201, Taiwan; ^7^Institute of Oral Sciences, Chung Shan Medical University, Taichung 40201, Taiwan

## Abstract

Caffeic acid phenethyl ester (CAPE), an active component extracted from honeybee hives, exhibits anti-inflammatory and anticancer activities. However, the molecular mechanism by which CAPE affects oral cancer cell metastasis has yet to be elucidated. In this study, we investigated the potential mechanisms underlying the effects of CAPE on the invasive ability of SCC-9 oral cancer cells. Results showed that CAPE attenuated SCC-9 cell migration and invasion at noncytotoxic concentrations (0 **μ**M to 40 **μ**M). Western blot and gelatin zymography analysis findings further indicated that CAPE downregulated matrix metalloproteinase-2 (MMP-2) protein expression and inhibited its enzymatic activity. CAPE exerted its inhibitory effects on MMP-2 expression and activity by upregulating tissue inhibitor of metalloproteinase-2 (TIMP-2) and potently decreased migration by reducing focal adhesion kinase (FAK) phosphorylation and the activation of its downstream signaling molecules p38/MAPK and JNK. These data indicate that CAPE could potentially be used as a chemoagent to prevent oral cancer metastasis.

## 1. Introduction

Oral cancer is associated with a high degree of local invasiveness and a high rate of metastasis to regional cervical lymph nodes. Oral squamous cell carcinoma (OSCC) is the most common head and neck cancer and is characterized by poor prognosis and low survival rate [1]. Metastasis is the major cause of cancer death and involves multiple processes including the remodeling and degradation of the extracellular matrix (ECM) [2]. Various proteinases, such as the matrix metalloproteinases (MMPs), cathepsins, and plasminogen activator (PA), are involved in the interactions between cancer cells and the ECM [3, 4]. The MMPs function in the degradation of structural proteins in the ECM and regulate several cellular functions including growth, invasion, metastasis, and angiogenesis [5]. Among the various MMPs thought to be involved in human disease, investigators have intensively investigated the gelatinases (MMP-2 and MMP-9). Matrix metalloproteinase-2 and MMP-9 are the principal enzymes involved in the degradation of the major structural component of the basement membrane, type IV collagen [6, 7]. Several studies have shown that the gelatinases are overexpressed in several types of human cancer, such as breast [8], pancreas [9], prostate [10], lung [11], and oral [12] cancers. Previous studies have also identified significantly increased urine and plasma MMP-2 concentrations in cancer patients [13, 14], with MMP-2 and MMP-9 being considered as predictors of risk or recurrence of metastasis or as cancer prognostic markers [15].

Caffeic acid phenethyl ester (CAPE) is one of the active components of honeybee propolis extracts [16]. Previous research has shown that CAPE exerts antioxidative, anti-inflammatory, and anticancer activities [17]. In addition, CAPE suppresses eicosanoid synthesis and inhibits the release of arachidonic acid from cell membranes [17, 18]. Its anti-inflammatory activity is derived from the downregulation of COX-2 expression [19, 20]. Chuu et al. identified that CAPE suppressed human prostate cancer cell proliferation through the inhibition of Akt [21]. Other studies have shown that CAPE induced apoptosis through the activation of Fas and Bax and the inhibition of NF-*κ*B [22, 23]. However, its effects on OSCC invasion and metastasis and their underlying mechanisms have yet to be fully elucidated.

This study investigated the association between CAPE treatment and invasion of SCC-9 oral cancer cells. Our results showed that CAPE suppressed the migration and invasion of oral cancer cells, probably through the inhibition of FAK phosphorylation and its downstream p38 and JNK signaling pathways. Results also indicated that the anti-invasive activity of CAPE is partly mediated by the regulation of MMP-2 expression and activity, which reduces the ability of oral cancer cells to degrade components of the extracellular matrix.

## 2. Materials and Methods

### 2.1. Cell Culture and CAPE Treatment

SCC-9, a human tongue squamous cell carcinoma cell line obtained from ATCC (Manassas, VA, USA), was cultured in Dulbecco's Modified Eagle's Medium supplemented with a nutrient mixture, which comprised F-12 Ham's medium (Life Technologies, Grand Island, NY, USA), 10% fetal bovine serum (Hyclone Laboratories, Logan, UT, USA), 2 mM glutamine, 100 U/mL penicillin, and 100 *μ*g/mL streptomycin. All cell cultures were maintained at 37°C in a humidified atmosphere of 5%  CO_2_. For CAPE treatment, appropriate amounts of stock solution of CAPE were added into culture medium to achieve the indicated concentrations and then incubated with cells for indicated time periods, whereas dimethyl sulfoxide solution without CAPE was used as blank reagent.

### 2.2. Determination of Cell Viability (MTT Assay)

For cell viability experiment, a microculture tetrazolium (3-(4,5-dimethylthiazol-2-yl)-2,5-diphenyltetrazolium bromide) colorimetric assay was performed to determine the cytotoxicity of CAPE [3]. SCC-9 cells were seeded in 24-well plates at a density of 5 × 10^4^ cells/well and treated with CAPE at a concentration between 0 and 40 *μ*M at 37°C for 24 and 48 h. After the exposure period, the media were removed, and cells were washed with phosphate-buffered saline (PBS) and then incubated with 20 *μ*L MTT (5 mg/mL) (Sigma chemical Co., St. Louis, MO, USA) for 4 h. The viable cell number per dish is directly proportional to the production of formazan, which can be measured spectrophotometrically at 563 nm following solubilization with isopropanol.

### 2.3. Annexin V/PI Double Staining

An FITC Annexin V Apoptosis Detection Kit I was used to quantify cell numbers in different stages of cell death. Briefly, 1 × 10^5^ cells were resuspended in 100 *μ*L 1x binding buffer (0.01 M Hepes/NaOH (pH 7.4), 0.14 M NaCl, and 2.5 mM CaCl_2_). With an addition of 5 *μ*L of FITC Annexin V and 5 *μ*L PI, the cell suspension was incubated for 15 minutes at room temperature in the dark. Afterwards, 400 *μ*L of 1x binding buffer was added to each tube followed by flow cytometry analysis within 1 hour.

### 2.4. In Vitro Wound Closure

SCC-9 cells (1 × 10^5^ cells/well) were plated in 6-well plates for 24 h, wounded by scratching with a pipette tip, then incubated with DMEM medium containing 0.5% FBS, and treated with or without CAPE (0, 5, 10, 20, and 40 *μ*M) for 0, 24, 48, and 72 h. Cells were photographed using a phase-contrast microscope (×100).

### 2.5. Cell Migration and Invasion Assays

Cell migration and invasion were assayed according to the methods described by Yang et al. [4]. After a treatment with CAPE (0, 5, 10, 20, and 40 *μ*M) for 24 h, surviving cells were harvested and seeded to Boyden chamber (Neuro Probe, Cabin John, MD, USA) at 10^4^ cells/well in serum-free medium and then incubated for 24 hours at 37°C. For invasion assay, 10 *μ*L Matrigel (25 mg/50 mL; BD Biosciences, MA, USA) was applied to 8 *μ*m pore-size polycarbonate membrane filters, and the bottom chamber contained standard medium. Filters were then air-dried for 5 h in a laminar flow hood. The invaded cells were fixed with 100% methanol and stained with 5% Giemsa. Cell numbers were counted under a light microscope. The migration assay was carried out as described in the invasion assay with no coating of Matrigel [3].

### 2.6. Determination of MMP-2 by Gelatin Zymography

The activities of MMP-2 in conditional medium were measured by gelatin zymography protease assays as previously described [3]. Briefly, collected media of an appropriate volume (adjusted by vital cell number) were prepared with SDS sample buffer without boiling or reduction and subjected to 0.1% gelatin and 8% SDS-PAGE electrophoresis. After electrophoresis, gels were washed with 2.5% Triton X-100 and then incubated in reaction buffer (40 mM Tris–HCl, pH 8.0, 10 mM CaCl_2_, and 0.01% NaN_3_) for 12 h at 37°C. Then the gel was stained with Coomassie Brilliant Blue R-250. 

### 2.7. Preparation of Total Cell Lysates

For total cell lysates preparation, cells were rinsed with PBS twice, scraped with 0.2 mL of cold RIPA buffer containing protease inhibitors cocktail, and then vortexed at 4°C for 10 min. Cell lysates were subjected to a centrifugation of 10,000 rpm for 10 min at 4°C, and the insoluble pellet was discarded. The protein concentration of total cell lysates and nuclear fraction was determined by Bradford assay [3]. 

### 2.8. Western Blot Analysis

The cell lysates were separated in a 10% polyacrylamide gel and transferred onto a nitrocellulose membrane. The blot was subsequently incubated with 5% nonfat milk in Tris-buffered saline (20 mM Tris, 137 mM NaCl, pH 7.6) for 1 h to block nonspecific binding and then incubated overnight with polyclonal antibodies against MMP-2, TIMP-2, caspase 3, 8, and 9, three MAPKs (ERK 1/2, JNK 1/2, and p38), Akt, or FAK with the specific antibodies for unphosphorylated or phosphorylated forms of the corresponding ERK 1/2, JNK 1/2, p38, Akt, and FAK. Blots were then incubated with a horseradish peroxidase goat anti-rabbit or anti-mouse IgG for 1 h. Afterwards, signal was detected by using enhanced chemiluminescence (ECL) commercial kit (Amersham Biosciences), and relative photographic density was quantitated by scanning the photographic negatives on a gel documentation and analysis system (AlphaImager 2000, Alpha Innotech Corporation, San Leandro, CA, USA).

### 2.9. Statistical Analysis

Statistical significances of difference throughout this study were calculated by Student's *t*-test (Sigma-Stat 2.0, Jandel Scientific, San Rafael, CA, USA). A difference at *P* < 0.05 was considered to be statistically significant, and the experiments were repeated three times. 

## 3. Results

### 3.1. Effects of CAPE on SCC-9 Cell Viability

**Figure 1:**
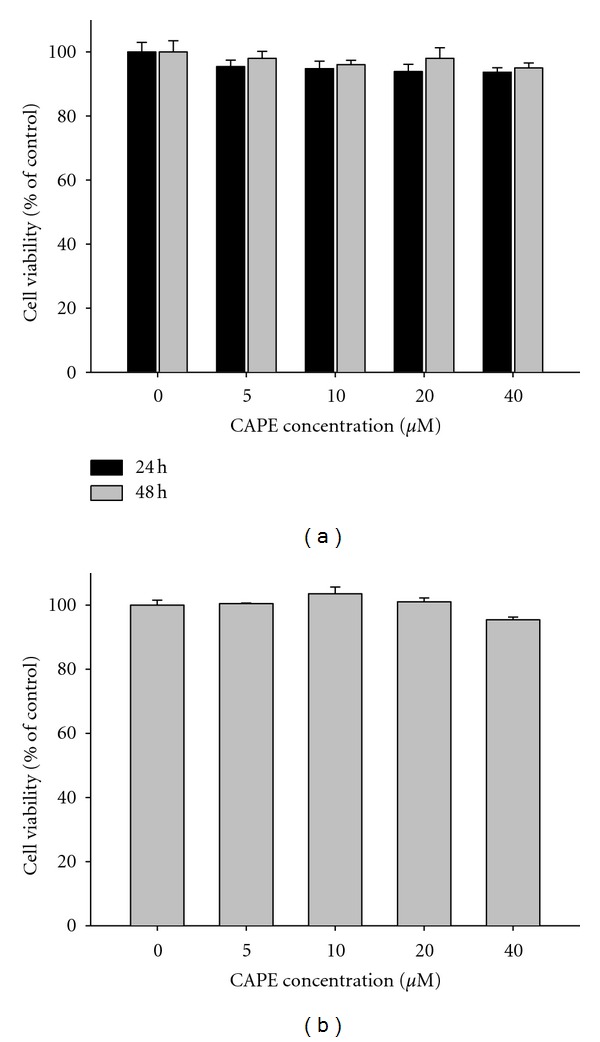
Effect of CAPE on cell viability. (a) SCC-9 cells were treated with CAPE (0, 5, 10, 20, and 40 *μ*M) for 24 h and 48 h before being subjected to an MTT assay for cell viability. (b) Normal gingival fibroblast cells were treated with CAPE (0, 5, 10, 20, and 40 *μ*M) for 24 h before being subjected to an MTT assay for cell viability. The values represented the means ± SD of at least three independent experiments.

Figure 1(a) displays the cytotoxic effects of various concentrations of CAPE (0 *μ*M–40 *μ*M) on SCC-9 cells for 24 hr and 48 hr. Results from MTT assay showed that all concentrations, including the highest tested CAPE concentration (40 *μ*M), had nonsignificant effects on SCC-9 cell viability compared to controls (*P* > 0.05). Furthermore, CAPE also did not alter the cell viability of normal gingival fibroblast at various concentrations (0–40 *μ*M) for 24 h (Figure 1(b)). Thus, all subsequent experiments, therefore, used this CAPE concentration range.

**Figure 2:**
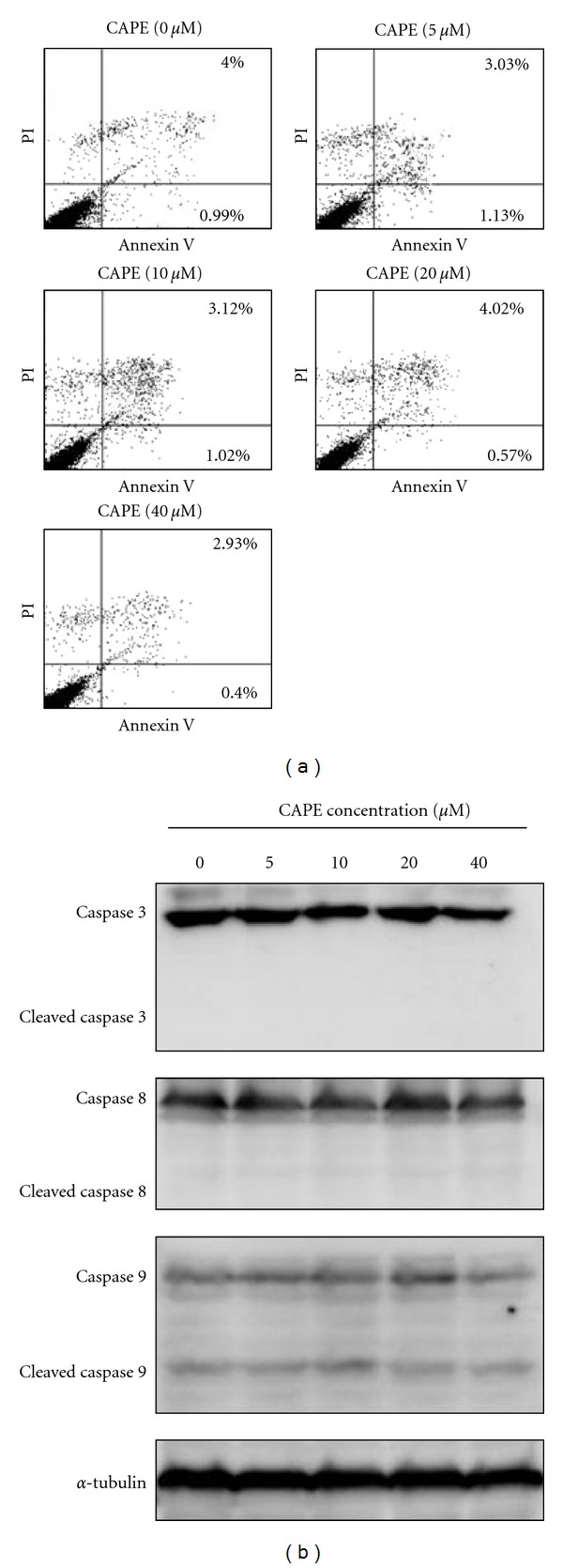
Effects of CAPE on the Annexin V flipping and caspase cleavage in SCC-9 cells. SCC-9 cells were treated with CAPE (0–40 *μ*M) for 24 h and then subjected to Annexin V and PI double-stained flow cytometry (a) or Western blotting to analyze the protein levels of caspase 3, 8, and 9 (b).

We further investigated the effect of CAPE on Annexin V flipping and caspase cleavage. Our results showed that treatment of SCC-9 cells with CAPE (0–40 *μ*M) for 24 h did not increase the incidence of apoptosis as evidenced without significant changes in the Annexin V/PI double staining (Figure 2(a)) and caspase 3, 8, and 9 expression levels (Figure 2(b)) between control and CAPE-treated SCC-9 cells (*P* > 0.05). 

### 3.2. Inhibition of SCC-9 Cell Motility, Migration, and Invasion by CAPE

**Figure 3:**
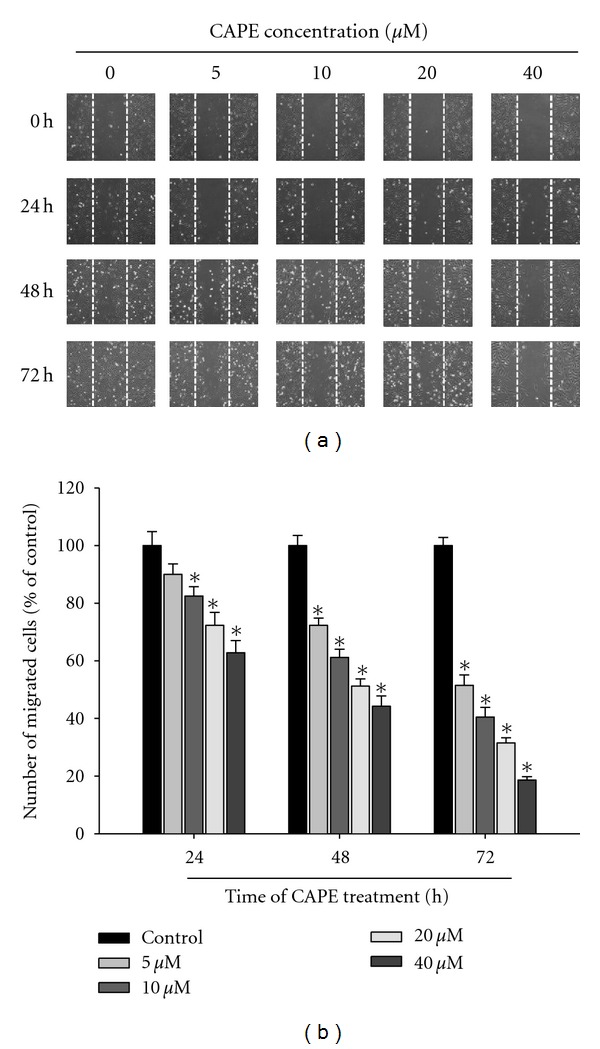
Effect of CAPE on in vitro wound closure in oral cancer cells. (a) SCC-9 cells were wounded and then treated with vehicle (DMSO) or CAPE (0, 5, 10, 20, and 40 *μ*M) for 0 h, 24 h, 48 h, and 72 h in 0.5% FBS-containing medium. At 0, 24 h, 48 and 72 h, phase-contrast pictures of the wounds at three different locations were taken. (b) Cells migrating into the wound area were counted using the dashed line as time zero. A quantitative assessment of the mean number of cells in the denuded zone is the mean ± SD (*n* = 3).

**Figure 4:**
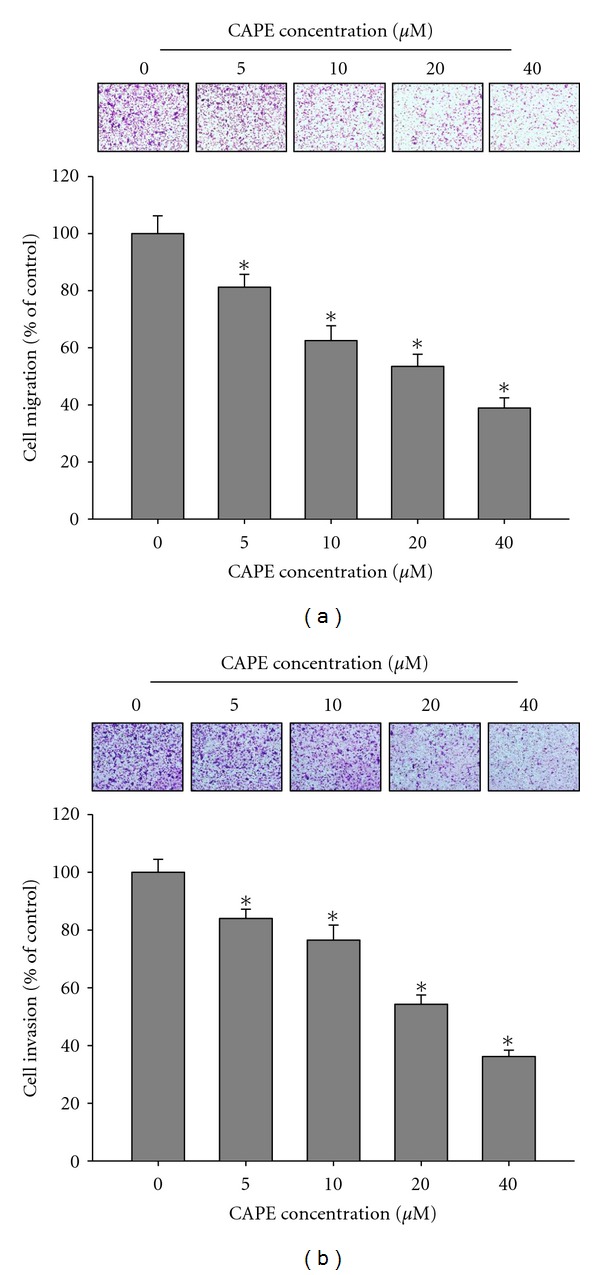
Effect of CAPE on cell migration and invasion in SCC-9 cells. (a) The cell migration and (b) cell invasion were measured using a Boyden chamber for 16 h and 24 h with polycarbonate filters, respectively. The migration and invasion abilities of SCC-9 cells were quantified by counting the number of cells that invaded to the underside of the porous polycarbonate as described in Section 2. The values represented the means ± SD of at least three independent experiments. *P < 0.05 as compared with the vehicle group.

 In the wound-healing assay, CAPE significantly reduced the cell motility of SCC-9 cells both dose and time dependently (*P* < 0.05) (Figures 3(a) and 3(b)). At 40 *μ*M, CAPE decreased the migrated cell number by 38%, 56%, and 82% at 24 h, 48 h, and 72 h, respectively. Using a cell migration and invasion assay with a Boyden chamber, we showed that CAPE reduced SCC-9 cell migration and invasion significantly and in a concentration-dependent manner (*P* < 0.05) (Figures 4(a) and 4(b)). In the invasion assay, the inhibition percentage was 16–64% (Figure 4(b)) after incubation of cells with different concentrations (0–40 *μ*M) of CAPE for 24 h. Our results indicated increased inhibition of cell migration with increasing CAPE concentration.

### 3.3. Effects of CAPE on MMP-2 and TIMP-2 Protein Expressions and MMP-2 Enzymatic Activity

**Figure 5:**
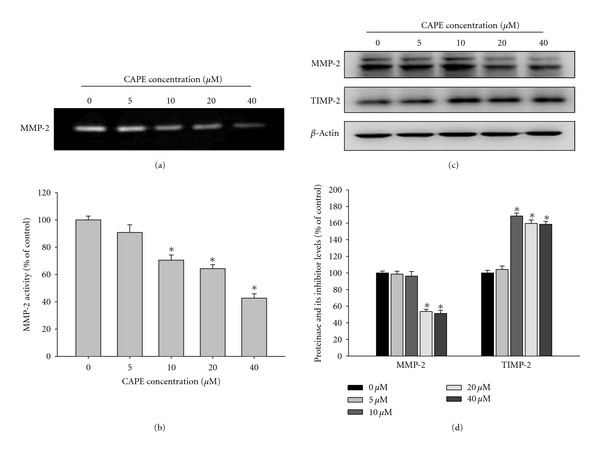
Effects of CAPE on the activity and protein level of MMP-2 and the protein level of the endogenous inhibitor TIMP-2. (a-b) SCC-9 cells were treated with CAPE (0, 5, 10, 20, and 40 *μ*M) for 24 h and then subjected to gelatin zymography to analyze the activity of MMP-2. (c-d) SCC-9 cells were treated with CAPE (0, 5, 10, 20, and 40 *μ*M) for 24 h and then subjected to western blotting to analyze the protein levels of MMP-2 and TIMP-2. Quantitative results of MMP-2 and TIMP-2 protein levels which were adjusted with *β*-actin protein level. The values represented the means ± SD of at least three independent experiments. *P < 0.05 as compared with the vehicle group.

 To confirm that MMP-2 plays a vital role in the suppression of cell migration, we compared MMP-2 protein expression in CAPE-treated and control SCC-9 cells. As shown in Figures 5(a) and 5(b), treatment of SCC-9 cells with CAPE downregulated MMP-2 enzymatic activity in a concentration-dependent manner (*P* < 0.05). At 40 *μ*M, CAPE decreased the MMP-2 enzymatic activity by 57% at 24 h. CAPE also significantly reduced MMP-2 protein expressions and increased TIMP-2 expression in western blotting (Figures 5(c) and 5(d)). CAPE (40 *μ*M) increased TIMP-2 expression with about 1.58 folds at 24 h.

### 3.4. Effects of CAPE on Focal Adhesion Kinase (FAK) Activation

**Figure 6:**
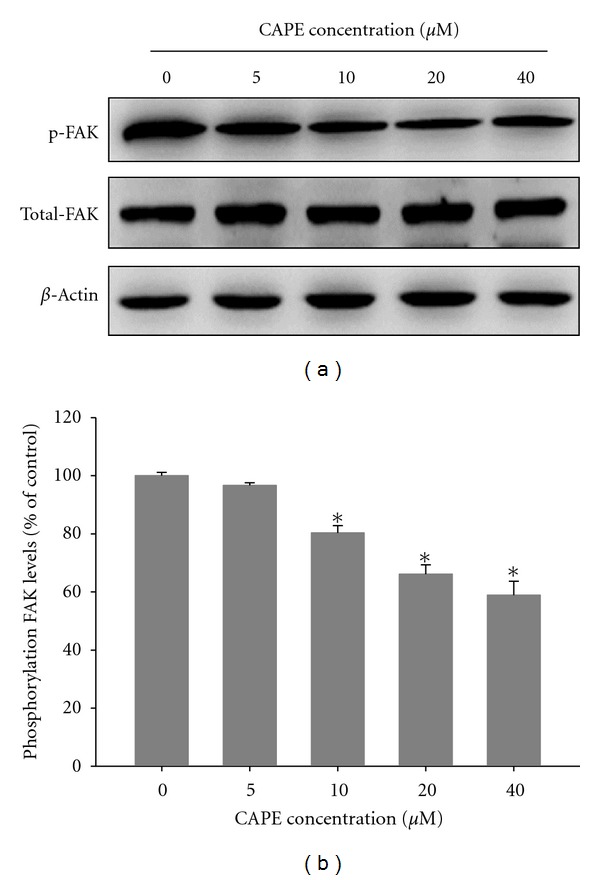
Effects of CAPE on the phosphorylation level of FAK. (a) SCC-9 cells were treated with CAPE (0, 5, 10, 20, and 40 *μ*M) for 24 h and then subjected to western blotting to analyze the levels of FAK. (b) Quantitative results of phosphorylation level of FAK which were adjusted with total FAK protein level. The values represented the means ± SD of at least three independent experiments. *P < 0.05 as compared with the vehicle group.

 Our investigation of the molecular regulation of cell migration indicated the involvement of FAK in CAPE activity. Having shown that CAPE inhibits SCC-9 cell migration and invasion, we further aimed to evaluate any changes in FAK activation in these cells using antibodies directed against the FAK phosphorylation site Tyr397. As shown in Figure 6, CAPE reduced FAK phosphorylation in SCC-9 cells. The inhibition percentage was 20–42% (Figure 6(b)) after incubation of cells with different concentrations of CAPE for 24 h. These results suggested that CAPE inhibits SCC-9 cell migration, at least in part, through the regulation of FAK phosphorylation.

### 3.5. Effects of CAPE on MAPK and PI3K/Akt Pathways

**Figure 7:**
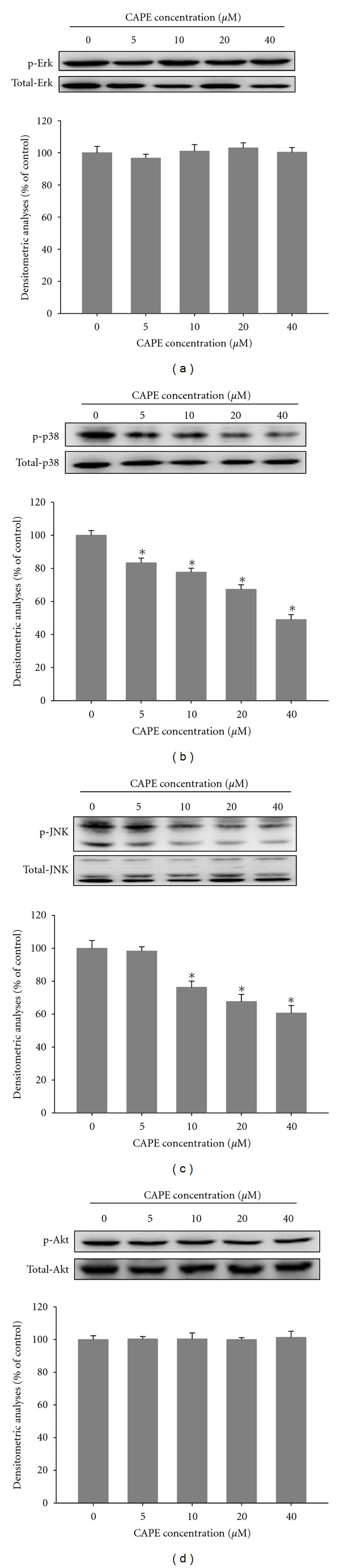
Effects of CAPE on the MAPKs pathway and Akt signalings. SCC-9 cells were cultured in various concentrations of CAPE (0, 5, 10, 20, and 40 *μ*M) for 24 hours, and then the cell lysates were subjected to SDS-PAGE followed by western blots with (a) anti-ERK1/2, (b) anti-p38, (c) anti-JNK, and (d) anti-Akt (total and phosphorylated) antibodies as described in Section 2. Determined activities of these proteins were subsequently quantified by densitometric analyses with that of control being 100% as shown just after the gel data. The values represented the means ± SD of at least 3 independent experiments. *P < 0.05 as compared with the vehicle group.

 We investigated the effects of CAPE on MAPK and PI3K/Akt pathways using western blot analysis. Results indicated the constitutive phosphorylation of pERK (Figure 7(a)) and pAKT (Figure 7(d)) in untreated SCC-9 cells and the downregulation of p38 (Figure 7(b)) and pJNK (Figure 7(c)) phosphorylation in CAPE-treated cells in a dose-dependent manner (*P* < 0.05). At 40 *μ*M, CAPE decreased phosphorylation levels of p38 and JNK by 51% and 40%, respectively.

## 4. Discussion

The management of patients with malignant tumors has improved greatly over the past few decades. Patients with OSCC now have an overall 5-year survival rate of approximately 25% [24]. However, patients with advanced disease often experience cancer spread to local and distant sites, which is poorly controlled by surgery. Our results suggested that CAPE could have potential use as an inhibitor of oral cancer metastasis and could, therefore, facilitate the development of effective anticancer therapies. We identified that CAPE (1) inhibits migration and invasion of SCC-9 oral cancer cells at noncytotoxic concentrations, (2) downregulates MMP-2 protein expression and inhibits its enzymatic activity, (3) increases TIMP-2 expression, (4) inhibits FAK phosphorylation, (5) and inhibits p38 MAPK and JNK activation.

Invasion and migration are considered the most important characteristics of malignant tumors. Solid tumor metastasis is the major cause of death in human cancer patients and involves multiple complicated processes. The degradation of the ECM is considered essential for tumor progression [25]. Among various proteases, which cause ECM degradation, matrix metalloproteinase-2 is the most significant in oral cancer [26]. Previous studies investigated the clinical significance of MMP-2 in different races with oral cancer. Immunohistochemical results indicated that MMP-2 expression significantly elevated in malignant tissues as compared with adjacent normal tissues. MMP-2 overexpression positively correlated with lymph node metastasis [27, 28]. The activation ratio of MMP-2 was higher in the malignant tissues of patients with lymph node metastasis as compared with those without lymph node metastasis [29]. OSCC invasion and metastasis represent major obstacles to treatment. Thus, inhibition of metastasis of OSCC by CAPE could provide important preventive and therapeutic benefitsagainst oral cancer.

The result of this study's showed that CAPE significantly inhibited the migratory/invasive ability of SCC-9 cancer cells, downregulated MMP-2 protein expression, and inhibited MMP-2 enzymatic activity. However, our data also indicated that the secreted concentrations of MMP-9 from SCC-9 cells were quite low (data not shown). The function of the MMP in vivo is dependent on the local balance between them and their natural inhibitors [30]. The TIMPs are physiological inhibitors that bind MMP in a 1 : 1 stoichiometry. Several studies have suggested that measuring the protease/protease inhibitor ratio in some tumors can provide an exact reflection of ECM remolding [31]. However, the relationship between metastasis and TIMP-2 remains controversial. In previous studies, TIMP-2 overexpression protected cancer cells from apoptosis and reduced cell invasion in various tumor cell lines [32, 33]. In other clinical analyses, TIMP-2 expression was positively associated with tumor recurrence [34]. Our data suggested a role for TIMP-2 in the prevention of tumor cell invasion because its expression is upregulated in CAPE-treated SCC-9 cells. 

Previous studies have well established the role of the Mitogen-activated protein kinase (MAPK) pathway in the regulation of MMP expression and metastasis of tumor cells [35–37]. Inhibition of p38 by SB203580 reduced the rate of migration of several cancer cells significantly [38]. In the study by Huang et al., the dominant-negative mutant of JNK inhibited the migration of human corneal epithelial cells [39]. In other studies, both p38 and JNK modulated MMP-2 production in cancer cells [40–42]. Our data showed that CAPE treatment inhibited p38 and JNK phosphorylation and downregulated MMP-2 expression, indicating a possible mechanism for the inhibition of MMP-2 synthesis by CAPE. The phosphoinositide-3 kinase (PI3K)/Akt signal transduction pathway is involved in MMP production and cell proliferation, survival, and migration [43]; however, our study results indicated that CAPE has nonsignificant effects on the PI3K/AKT signaling pathway.

In conclusion, the result of our study suggested that CAPE partly exerts its antimetastatic effects on oral cancer cells by regulating MMP-2 expression through the inhibition of FAK and MAPK activation. Overall, these data suggest that CAPE has a potential use as a chemoagent for the prevention of oral cancer cell metastasis.

## References

[1] Spiro RH, Alfonso AE, Farr HW, Strong EW (1974). Cervical node metastasis from epidermoid carcinoma of the oral cavity and oropharynx. A critical assessment of current staging. *American Journal of Surgery*.

[2] Stetler-Stevenson WG, Liotta LA, Kleiner DE (1993). Extracellular matrix 6: role of matrix metalloproteinases in tumor invasion and metastasis. *FASEB Journal*.

[3] Chien MH, Ying TH, Hsieh YS (2012). Dioscorea nipponica Makino inhibits migration and invasion of human oral cancer HSC-3 cells by transcriptional inhibition of matrix metalloproteinase-2 through modulation of CREB and AP-1 activity. *Food and Chemical Toxicology*.

[4] Yang SF, Chen MK, Hsieh YS (2010). Antimetastatic effects of *Terminalia catappa* L. on oral cancer via a down-regulation of metastasis-associated proteases. *Food and Chemical Toxicology*.

[5] Egeblad M, Werb Z (2002). New functions for the matrix metalloproteinases in cancer progression. *Nature Reviews Cancer*.

[6] Nelson AR, Fingleton B, Rothenberg ML, Matrisian LM (2000). Matrix metalloproteinases: biologic activity and clinical implications. *Journal of Clinical Oncology*.

[7] Björklund M, Koivunen E (2005). Gelatinase-mediated migration and invasion of cancer cells. *Biochimica et Biophysica Acta*.

[8] Ranuncolo SM, Armanasco E, Cresta C, De Kier Joffe EB, Puricelli L (2003). Plasma MMP-9 (92 kDa-MMP) activity is useful in the follow-up and in the assessment of prognosis in breast cancer patients. *International Journal of Cancer*.

[9] Yokoyama M, Ochi K, Ichimura M (2002). Matrix metalloproteinase-2 in pancreatic juice for diagnosis of pancreatic cancer. *Pancreas*.

[10] Sauer CG, Kappeler A, Späth M (2004). Expression and activity of matrix metalloproteinases-2 and -9 in serum, core needle biopsies and tissue specimens of prostate cancer patients. *Virchows Archiv*.

[11] Bugdayci G, Kaplan T, Sezer S (2006). Matrix metalloproteinase-9 in broncho-alveolar lavage fluid of patients with non-small cell lung cancer. *Experimental Oncology*.

[12] Hong SD, Hong SP, Lee JI, Lim CY (2000). Expression of matrix metalloproteinase-2 and -9 in oral squamous cell carcinomas with regard to the metastatic potential. *Oral Oncology*.

[13] Coticchia CM, Curatolo AS, Zurakowski D (2011). Urinary MMP-2 and MMP-9 predict the presence of ovarian cancer in women with normal CA125 levels. *Gynecologic Oncology*.

[14] Wong TS, Kwong DLW, Sham JST, Wei WI, Kwong YL, Yuen APW (2004). Clinicopathologic significance of plasma matrix metalloproteinase-2 and -9 levels in patients with undifferentiated nasopharyngeal carcinoma. *European Journal of Surgical Oncology*.

[15] Song N, Sung H, Choi JY (2012). Preoperative serum levels of matrix metalloproteinase-2 (MMP-2) and survival of breast cancer among Korean women. *Cancer Epidemiology, Biomarkers & Prevention*.

[16] Dobrowolski JW, Vohora SB, Sharma K, Shah SA, Naqvi SAH, Dandiya PC (1991). Antibacterial, antifungal, antiamoebic, antiinflammatory and antipyretic studies on propolis bee products. *Journal of Ethnopharmacology*.

[17] Michaluart P, Masferrer JL, Carothers AM (1999). Inhibitory effects of caffeic acid phenethyl ester on the activity and expression of cyclooxygenase-2 in human oral epithelial cells and in a rat model of inflammation. *Cancer Research*.

[18] Mirzoeva OK, Yaqoob P, Knox KA, Calder PC (1996). Inhibition of ICE-family cysteine proteases rescues murine lymphocytes from lipoxygenase inhibitor-induced apoptosis. *FEBS Letters*.

[19] Lee Y, Shin DH, Kim JH (2010). Caffeic acid phenethyl ester-mediated Nrf2 activation and I*κ*B kinase inhibition are involved in NF*κ*B inhibitory effect: structural analysis for NF*κ*B inhibition. *European Journal of Pharmacology*.

[20] Jung WK, Lee DY, Choi YH (2008). Caffeic acid phenethyl ester attenuates allergic airway inflammation and hyperresponsiveness in murine model of ovalbumin-induced asthma. *Life Sciences*.

[21] Chuu CP, Lin HP, Ciaccio MF (2012). Caffeic acid phenethyl ester suppresses the proliferation of human prostate cancer cells through inhibition of p70S6K and Akt signaling networks. *Cancer Prevention Research*.

[22] Lee YT, Don MJ, Hung PS (2005). Cytotoxicity of phenolic acid phenethyl esters on oral cancer cells. *Cancer Letters*.

[23] Watabe M, Hishikawa K, Takayanagi A, Shimizu N, Nakaki T (2004). caffeic acid phenethyl ester induces apoptosis by inhibition of
NFkappaB and activation of Fas in human breast cancer MCF-7 cells. *Journal of Biological Chemistry*.

[24] Ramqvist T, Dalianis T (2010). Oropharyngeal cancer epidemic and human papillomavirus. *Emerging Infectious Diseases*.

[25] Coussens LM, Werb Z (1996). Matrix metalloproteinases and the development of cancer. *Chemistry and Biology*.

[26] Chien MH, Lin CW, Cheng CW, Wen YC, Yang SF Matrix metalloproteinase (MMP)-2 as a target for head and neck cancer therapy.

[27] Katayama A, Bandoh N, Kishibe K (2004). Expressions of matrix metalloproteinases in early-stage oral squamous cell carcinoma as predictive indicators for tumor metastases and prognosis. *Clinical Cancer Research*.

[28] Kurahara S, Shinohara M, Ikebe T (1999). Expression of MMPS, MT-MMP, and TIMPs in squamous cell carcinoma of the oral cavity: correlations with tumor invasion and metastasis. *Head & Neck*.

[29] Qin H, Sun Y, Benveniste EN (1999). The transcription factors Sp1, Sp3, and AP-2 are required for constitutive matrix metalloproteinase-2 gene expression in astroglioma cells. *Journal of Biological Chemistry*.

[30] Snoek-van Beurden PAM, Von Den Hoff JW (2005). Zymographic techniques for the analysis of matrix metalloproteinases and their inhibitors. *BioTechniques*.

[31] Nuttall RK, Pennington CJ, Taplin J (2003). Elevated membrane-type matrix metalloproteinases in gliomas revealed by profiling proteases and inhibitors in human cancer cells. *Molecular Cancer Research*.

[32] Imren S, Kohn DB, Shimada H, Blavier L, DeClerck YA (1996). Overexpression of tissue inhibitor of metalloproteinases-2 by retroviral-mediated gene transfer in vivo inhibits tumor growth and invasion. *Cancer Research*.

[33] Valente P, Fassina G, Melchiori A (1998). TIMP-2 over-expression reduces invasion and angiogenesis and protects B16F10 melanoma cells from apoptosis. *International Journal of Cancer*.

[34] Visscher DW, Höyhtyä M, Ottosen SK (1994). Enhanced expression of tissue inhibitor of metalloproteinase-2 (TIMP-2) in the stroma of breast carcinomas correlates with tumor recurrence. *International Journal of Cancer*.

[35] Chen PN, Hsieh YS, Chiou HL, Chu SC (2005). Silibinin inhibits cell invasion through inactivation of both PI3K-Akt and MAPK signaling pathways. *Chemico-Biological Interactions*.

[36] Hsieh YS, Chu SC, Yang SF, Chen PN, Liu YC, Lu KH (2007). Silibinin suppresses human osteosarcoma MG-63 cell invasion by inhibiting the ERK-dependent c-Jun/AP-1 induction of MMP-2. *Carcinogenesis*.

[37] Weng CJ, Chau CF, Hsieh YS, Yang SF, Yen GC (2008). Lucidenic acid inhibits PMA-induced invasion of human hepatoma cells through inactivating MAPK/ERK signal transduction pathway and reducing binding activities of NF-*κ*B and AP-1. *Carcinogenesis*.

[38] Huang C, Jacobson K, Schaller MD (2004). MAP kinases and cell migration. *Journal of Cell Science*.

[39] Huang Z, Yan DP, Ge BX (2008). JNK regulates cell migration through promotion of tyrosine phosphorylation of paxillin. *Cellular Signalling*.

[40] Fromigué O, Hamidouche Z, Marie PJ (2008). Blockade of the RhoA-JNK-c-Jun-MMP2 cascade by atorvastatin reduces osteosarcoma cell invasion. *Journal of Biological Chemistry*.

[41] Zhong J, Gencay MMC, Bubendorf L (2006). ERK1/2 and p38 MAP kinase control MMP-2, MT1-MMP, and TIMP action and affect cell migration: a comparison between mesothelioma and mesothelial cells. *Journal of Cellular Physiology*.

[42] Kumar B, Koul S, Petersen J (2010). p38 Mitogen-activated protein kinase-driven MAPKAPK2 regulates invasion of bladder cancer by modulation of MMP-2 and MMP-9 activity. *Cancer Research*.

[43] Tian T, Nan KJ, Guo H (2010). PTEN inhibits the migration and invasion of HepG2 cells by coordinately decreasing MMP expression via the PI3K/Akt pathway. *Oncology Reports*.

