# Corrigendum to “Caffeic Acid Phenethyl Ester Inhibits Oral Cancer Cell Metastasis by Regulating Matrix Metalloproteinase-2 and the Mitogen-Activated Protein Kinase Pathway”

**DOI:** 10.1155/2016/6728642

**Published:** 2016-06-28

**Authors:** Chih-Yu Peng, Hui-Wen Yang, Yin-Hung Chu, Yu-Chao Chang, Ming-Ju Hsieh, Ming-Yung Chou, Kun-Tu Yeh, Yueh-Min Lin, Shun-Fa Yang, Chiao-Wen Lin

**Affiliations:** ^1^School of Dentistry, Chung Shan Medical University, Taichung 40201, Taiwan; ^2^Department of Dentistry, Chung Shan Medical University Hospital, Taichung 40201, Taiwan; ^3^Institute of Medicine, Chung Shan Medical University, Taichung 402, Taiwan; ^4^School of Medical Laboratory and Biotechnology, Chung Shan Medical University, Taichung 40201, Taiwan; ^5^Department of Pathology, Changhua Christian Hospital, Changhua 50006, Taiwan; ^6^Department of Medical Research, Chung Shan Medical University Hospital, Taichung 40201, Taiwan; ^7^Institute of Oral Sciences, Chung Shan Medical University, Taichung 40201, Taiwan

We have noticed an inadvertent error in our paper “Caffeic Acid Phenethyl Ester Inhibits Oral Cancer Cell Metastasis by Regulating Matrix Metalloproteinase-2 and the Mitogen-Activated Protein Kinase Pathway” [1].

We found a misplaced figure in Figure 3(a). We have attached a corrected version of Figure 3(a). The correction does not affect the findings or conclusion of the study.

## Figures and Tables

**Figure 3:**
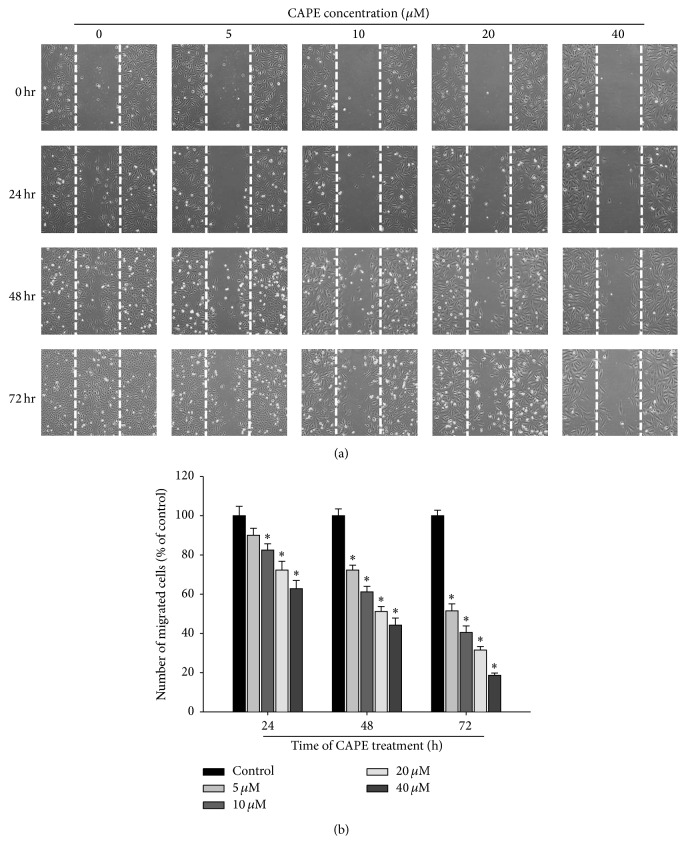
Effect of CAPE on in vitro wound closure in oral cancer cells. (a) SCC-9 cells were wounded and then treated with vehicle (DMSO) or CAPE (0, 5, 10, 20, and 40 *μ*M) for 0 h, 24 h, 48 h, and 72 h in 0.5% FBS-containing medium. At 0 h, 24 h, 48, and 72 h, phase-contrast pictures of the wounds at three different locations were taken. (b) Cells migrating into the wound area were counted using the dashed line as time zero. A quantitative assessment of the mean number of cells in the denuded zone is the mean ± SD (*n* = 3).
